# *Maclura cochinchinensis* (Lour.) Corner Heartwood Extracts Containing Resveratrol and Oxyresveratrol Inhibit Melanogenesis in B16F10 Melanoma Cells

**DOI:** 10.3390/molecules29112473

**Published:** 2024-05-24

**Authors:** Worrawat Promden, Pithi Chanvorachote, Wittawat Viriyabancha, Siriluk Sintupachee, Wanchai De-Eknamkul

**Affiliations:** 1 Division of General Science, Faculty of Education, Buriram Rajabhat University, Buriram 31000, Thailand; 2 Center of Excellence in Cancer Cell and Molecular Biology, Faculty of Pharmaceutical Sciences, Chulalongkorn University, Bangkok 10330, Thailand; pithi.c@chula.ac.th; 3 Department of Pharmacology and Physiology, Faculty of Pharmaceutical Sciences, Chulalongkorn University, Bangkok 10330, Thailand; 4 Medicines Regulation Division, Food and Drug Administration, Ministry of Public Health, Nonthaburi 11000, Thailand; wittawat.v@fda.moph.go.th; 5 Program in Creative Innovation in Science and Technology, Faculty of Science and Technology, Nakhon Si Thammarat Rajabhat University, Nakhon Si Thammarat 80280, Thailand; siriluk_sint@nstru.ac.th; 6 Specialized Research Unit for Insects and Herbs, Nakhon Si Thammarat Rajabhat University, Nakhon Si Thammarat 80280, Thailand; 7 Natural Product Biotechnology Research Unit, Department of Pharmacognosy and Pharmaceutical Botany, Faculty of Pharmaceutical Sciences, Chulalongkorn University, Bangkok 10330, Thailand; wanchai.d@chula.ac.th

**Keywords:** *Maclura cochinchinensis*, anti-tyrosinase, melanogenesis, mouse melanoma B16F10, oxyresveratrol, resveratrol, HPTLC, HPLC, LC-MS/MS

## Abstract

This study aimed to isolate and purify resveratrol and oxyresveratrol from the heartwoods of *Maclura cochinchinensis*, and to evaluate their inhibitory effects on melanogenesis in B16F10 murine melanoma cells. A methanol maceration process yielded a crude extract comprising 24.86% of the initial mass, which was subsequently analyzed through HPTLC, HPLC, and LC-MS/MS. These analyses revealed the presence of resveratrol and oxyresveratrol at concentrations of 4.32 mg/g and 33.6 mg/g in the extract, respectively. Initial purification employing food-grade silica gel column chromatography separated the extract into two fractions: FA, exhibiting potent inhibition of both tyrosinase activity and melanogenesis, and FM, showing no such inhibitory activity. Further purification processes led to the isolation of fractions Y11 and Gn12 with enhanced concentrations of resveratrol (94.9 and 110.21 mg/g, respectively) and fractions Gn15 and Gn16 with elevated levels of oxyresveratrol (321.93 and 274.59 mg/g, respectively), all of which significantly reduced melanin synthesis. These outcomes affirm the substantial presence of resveratrol and oxyresveratrol in the heartwood of *M. cochinchinensis*, indicating their promising role as natural agents for skin lightening.

## 1. Introduction

Tyrosinase, a crucial oxidase in melanogenesis within human melanocytes and enzymatic browning in foods, facilitates the conversion of tyrosine to L-DOPA and its subsequent oxidation, leading to melanin production. This pigment is essential for determining skin coloration, hair, and other tissues [1,2]. However, its overproduction can cause various hyperpigmentation disorders, necessitating control over tyrosinase activity to prevent such conditions [3,4]. While the cosmeceutical industry has developed several synthetic whitening agents, these often entail adverse effects such as erythema and dermatitis upon prolonged use [5]. In contrast, plant-derived tyrosinase inhibitors are emerging as favorable options for skin whitening due to their lower toxicity and higher bioavailability, making them suitable for food and cosmetic use [6]. Indeed, the tyrosinase inhibitory potential of numerous indigenous plants has been documented globally, positioning natural plant extracts as promising alternatives for safer medical cosmetics.

Thailand, situated in the Indo-Burma region, is one of the world’s leading biodiversity hotspots. It harbors over 15,000 plant species, many of which are integral to traditional folk medicine practices [7,8]. Among these, *Maclura cochinchinensis* (Lour.), a spiny shrub or woody climber from the Moraceae family, commonly known as cockspur thorn or “Kae Lae” in Thai, stands out for its medicinal use [9,10,11,12]. Found across several Asian countries, including China, Japan, and India, *M. cochinchinensis* is utilized in Thai traditional medicine for treating a range of conditions, from chronic fever and skin infections to diabetes and lymph node abnormalities [10,13,14]. The heartwood of this plant is rich in chemical constituents like morin, resveratrol, and oxyresveratrol, contributing to its anti-inflammatory, antioxidant, and antimicrobial activities [13,15,16]. The plant’s extracts, particularly from the heartwood, have also been demonstrated to exhibit strong antioxidant and anti-tyrosinase activities [17,18], suggesting potential use in cosmeceutical products.

Extraction methods for resveratrol have evolved to enhance yield and maintain bioactivity. Techniques such as microbial fermentation, enzyme-assisted extraction, and ultrasonic extraction have been employed, achieving high yields of natural resveratrol while protecting the ecological environment [19,20,21]. Oxyresveratrol, a resveratrol derivative, has been the subject of various extraction studies due to its diverse biological activities and pharmacological benefits. The extraction of oxyresveratrol from different sources, including *Morus alba* L. and *Artocarpus lakoocha* heartwood, has been optimized through several methods to enhance yield and efficiency. Microwave-assisted extraction (MAE) of mulberry twigs using surfactants such as Tween 80 and Tween 20 was explored but showed lower oxyresveratrol content compared to traditional maceration methods. This indicates the need for further optimization of extraction conditions, including temperature, pH, and microwave energy. [22]. Conversely, maceration using 70-percent ethanol for 6 h extracted a significant amount of oxyresveratrol from *A. lakoocha*, demonstrating its effectiveness and also highlighting the antioxidant capacity of the extract [23].

Since *M. cochinchinensis* is a significant source of resveratrol and oxyresveratrol, this study employed maceration to extract these compounds from the heartwood. This method avoids high temperatures that can affect compound stability. A serial exhaustive extraction using a range of solvents from nonpolar to polar was conducted to select the most appropriate solvents for enriching target compounds and removing impurities. The extracts were initially assessed for mushroom tyrosinase inhibition, followed by the use of column chromatography fractionation to further separate and purify the extracts. HPTLC analysis was then employed to detect resveratrol and oxyresveratrol in each fraction. Subsequent assays evaluated the extracts for anti-tyrosinase and melanogenesis inhibition, antioxidant activity against DPPH radicals, and cytotoxicity in HaCaT, HDFn, and B16F10 cells. Resveratrol and oxyresveratrol were analyzed both quantitatively and qualitatively via HPLC and LC-MS/MS. Additionally, a cost-effective method of column chromatography fractionation was introduced, achieving better separation and purification of the heartwood extract.

## 2. Results and Discussion

### 2.1. Extraction Yields and Screening for Anti-Tyrosinase Activities

The extraction of plant active ingredients is a critical process comprising steps such as extraction, separation, concentration, and drying, with extraction being fundamental to determining the product’s quality and cost. Traditional extraction methods—decoction, maceration, reflux, and Soxhlet—are prevalent for phytochemical isolation from plants [24,25]. Among solvent extraction techniques, including low-temperature extraction, thermal dissolution, and ionic liquid extraction, low-temperature extraction is favored for isolating thermolabile compounds due to its effectiveness and prevalence [26]. This study adopts maceration through serial exhaustive extraction from nonpolar to polar solvents to isolate bioactive compounds from *M. cochinchinensis* heartwood, circumventing the application of heat.

The systematic examination of serial exhaustive extraction from *M. cochinchinensis* heartwoods using a solvent polarity gradient yielded fifteen extracts. These extracts demonstrated variable yields from 0 mg to 1460 mg, corresponding to percentage yields ranging from 0% to 14.6%, dependent on the solvent utilized (Table 1). The employment of hexane as a solvent resulted in minimal yields (0% to 2.4%), indicating a sparse presence of non-polar bioactive compounds within the heartwoods. In contrast, methanol proved to be the most effective solvent, producing a yield of 1460 mg, or 14.6%. Methanolic extracts were characterized by their dark brown hue and the presence of dense, viscous substances that emitted a distinctive aroma reminiscent of burning wood.

The previous study on extracting bioactive compounds from *M. cochinchinensis* heartwood utilized various methods, leading to different yields. Sato et al. achieved the highest yield of 30.2% through 24 h maceration using 70% ethanol or ethyl acetate [10]. Chewchinda et al. reported a yield of 18.99 ± 0.65% *w*/*w* after boiling dried heartwood powder in distilled water, followed by filtration and spray drying of the filtrate [13]. Furthermore, Rueankham et al. determined that ethanol extraction produced the highest yields, ranging from 5.18% to 9.56% when examining the effects of solvents of varying polarities [16]. These outcomes highlight the crucial influence of solvent selection on the efficiency of extracting bioactive compounds from *M. cochinchinensis* heartwood, emphasizing the significance of strategic solvent choice to enhance yields.

In this study, as illustrated in Table 1, the extracts with an extraction yield above 0% were screened for the potential to inhibit mushroom tyrosinase at a 2.5-µg/mL concentration. The extracts exhibited anti-tyrosinase activities ranging from undetected to 83.64%, with IC_50_ ranging from undetected to 6.90 µg/mL. As expected, no tyrosinase inhibitory activity was observed for the hexane extracts, while the methanolic extracts (H_2_, A_1_, M_0_, and W_1_) possessed maximum anti-tyrosinase activity of 78.53–83.64% with an IC_50_ range of 0.12–0.28 µg/mL. The capability of the methanolic extracts to inhibit mushroom tyrosinase was up to 115-fold greater than that of kojic acid (IC_50_ = 13.85 µg/mL) but 6-fold lower than that of oxyresveratrol (IC_50_ = 0.02 µg/mL). In the previous studies, extracts from the heartwood of *M. cochinchinensis* were obtained using ultrasonic extraction and demonstrated significant anti-tyrosinase properties. The IC_50_ values were 7.60 μg/mL for the 80% ethanol extract and 7.91 μg/mL for the methanol extract. Oxyresveratrol, not resveratrol, was identified as a potent bioactive compound contributing to this inhibitory effect [18].

According to the results, methanol was chosen for the scale-up of extraction processes in subsequent investigations due to its demonstrated effectiveness. Resveratrol and oxyresveratrol, compounds recognized for their substantial bioactivity and value, significantly contributed to these findings. Consequently, this study underscores the pronounced efficacy of methanolic extracts from *M. cochinchinensis* in effectively inhibiting tyrosinase, thereby enabling further development of research methodologies for enhanced separation and purification of resveratrol and oxyresveratrol.

### 2.2. Stepwise Column Chromatography Fractionation and Resveratrol and Oxyresveratrol Contents

To obtain resveratrol and oxyresveratrol enriched extract, the plant crude extract was further cleaned up by a packed column using the nonpolar to polar solvent system as shown in Table 2 and Table 3. Since analytical-grade silica gel is costly, this study introduced food-grade silica gel in the first-step column chromatography, and analytical-grade silica gel was still used in the second step.

**Figure 1:**
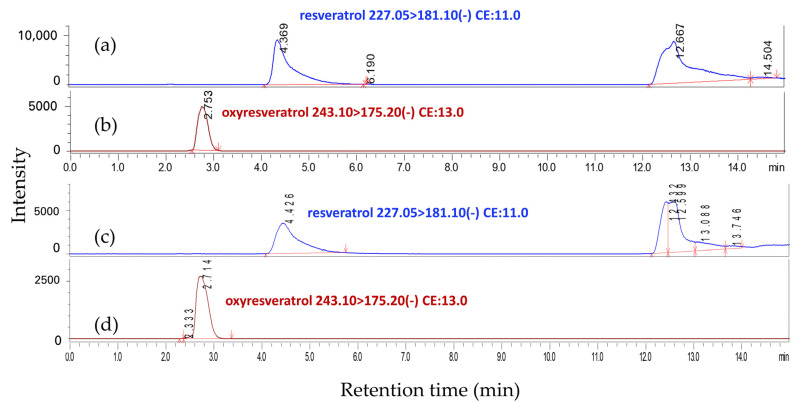
MRM LC-MS/MS chromatograms of standard resveratrol (**a**) and oxyresveratrol (**b**) compared to the extract from *M. cochinchinensis* fraction FA (**c**,**d**).

**Figure 2:**
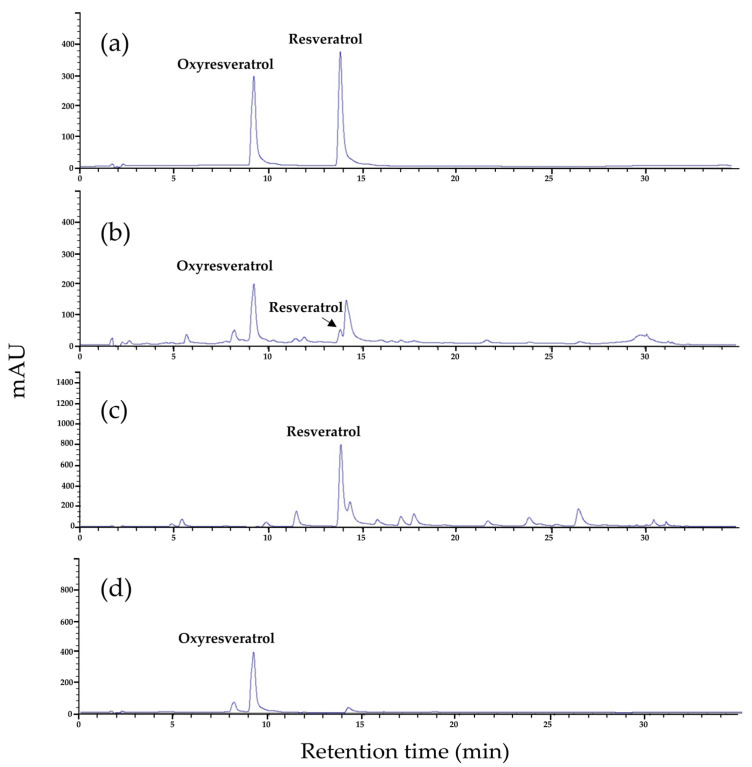
HPLC chromatograms at a wavelength of 320 nm of standard resveratrol and oxyresveratrol (**a**), the extract from *M. cochinchinensis* fraction FA (**b**), Fraction Y11 (**c**), and fraction Gn15 (**d**).

**Figure 3:**
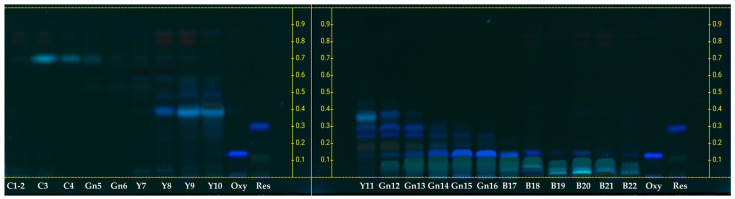
HPTLC profiles for fractions C1-2 through B22, each loaded with 5 µg of sample, alongside 2 µg of standard oxyresveratrol (Oxy, R_f_ ≈ 0.12) and 2 µg of standard resveratrol (Res, R_f_ ≈ 0.30), analyzed under a wavelength of 366 nm.

As presented in Table 4, the first-step column chromatography yielded two major fractions: FA (48.46 g) and FM (13.68 g). The FA fraction was described as having a yellow appearance, whereas the FM fraction was noted for its dark brown color and the presence of dense, viscous materials. LC-MS/MS analyses substantiated the existence of resveratrol and oxyresveratrol in fraction FA. The detection of both compounds was achieved through multiple reaction monitoring (MRM), which observed the transitions from precursor ions (in negative ionization mode) to product ions with mass-to-charge ratios (*m*/*z*) of 227.05 to 181.1 and 243.1 to 175.2, respectively, as illustrated in Figure 1 and Table 5. Quantitative assessment via HPLC revealed that fraction FA comprised 312.57 mg of resveratrol and 2441.90 mg of oxyresveratrol, corresponding to 0.65% and 5.04% of the total composition of the fraction, respectively. These findings are detailed in Table 4 and illustrated in the HPLC chromatogram presented in Figure 2b. In contrast, fraction FM was found to contain significantly lower concentrations of resveratrol and oxyresveratrol, while a high concentration of morin was detected within this fraction through HPLC analysis. Consequently, fraction FM was not selected for subsequent purification processes, and only fraction FA was advanced to the next step of purification. Subsequent fractionation of FA during the second purification step yielded 22 distinct fractions, as detailed in Table 5. Initial screening for resveratrol and oxyresveratrol was performed at a detection wavelength of 327 nm, where fractions Y7 to Y9 and Y10 to B17 exhibited the highest absorbance levels. HPTLC analysis was subsequently conducted to verify the presence of these compounds. This analysis confirmed that resveratrol was present in fractions Y11 to Gn13, and oxyresveratrol was identified in fractions Gn13 to B17, as depicted in Figure 3.

The quantification of resveratrol in fractions Y10 to Gn13, as determined by HPLC, varied from 0.05 mg to 114.83 mg, translating to concentrations ranging from 0.22 mg/g to 110.21 mg/g. Similarly, the oxyresveratrol content in fractions Gn13 to B17 was found to span from 62.05 mg to 1078.47 mg, corresponding to concentrations between 34.86 mg/g and 321.93 mg/g (Table 4). Figure 2c,d display the chromatograms of fractions Y11 and Gn15, respectively, showcasing the presence of resveratrol and oxyresveratrol.

The high recovery of resveratrol and oxyresveratrol could be attributed to the weaker interaction between these compounds and the packing materials in the solvent system. Thus, both compounds were likely to elute from the column along with the mobile phase rather than adsorb onto the surface of the packed materials. Possibly, the polarity of resveratrol is also closer to the 60% hexane/40% ethyl acetate eluent system, while that of oxyresveratrol is closer to the 50% hexane/50% ethyl acetate eluent system. This is because higher hexane (>60%, *v*/*v*) and ethyl acetate (>50%, *v*/*v*) concentrations did not improve the recovery of resveratrol and oxyresveratrol significantly. It is well documented that resveratrol is a weak acid consisting of two phenols joined by a double bond to form 3,5,4′-trihydroxy-trans-stilbene. It is not soluble in water but is highly soluble in alcohol and PEG-400 [27]. On the other hand, oxyresveratrol is a weak acid with four hydroxyl groups known as polyhydroxystilbene. This compound is dissolved well in organic solvents, for example, ethanol, dimethyl sulfoxide (DMSO), and dimethylformamide (DMF) [28].

### 2.3. Anti-Mushroom Tyrosinase Activity

The fractions derived from column chromatography fractionation were tested for anti-tyrosinase activities and melanogenesis inhibition. The results revealed that the capabilities of fractions FA and FM obtained from the first step of column chromatography fractionation were strikingly different (Table 4). Fraction FA possessed high tyrosinase inhibitory activity (IC_50_ = 0.25 µg/mL) and could suppress melanogenesis in B16F10 cells by 54.41% at a concentration of 25 µg/mL. Meanwhile, fraction FM had a low potential to inhibit mushroom tyrosinase activity and melanogenesis. The fractions obtained from the second-step column chromatography were also determined for the tyrosinase inhibition activities and melanogenesis suppression. The results showed that all the fractions tested displayed mushroom tyrosinase inhibitory activities, with pronounced inhibitory levels observed in fractions Gn13 (IC_50_ = 0.096 µg/mL), Gn14 (IC_50_ = 0.052 µg/mL), and Gn15 (IC_50_ = 0.045 µg/mL). In this study, it has been observed that fractions enriched with oxyresveratrol exhibited markedly stronger inhibitory effects against mushroom tyrosinase compared to those containing resveratrol. Among the compounds evaluated for their potential to inhibit mushroom tyrosinase, oxyresveratrol and resveratrol have emerged as compounds with significant inhibitory properties. Notably, oxyresveratrol has shown a pronounced capability to inhibit mushroom tyrosinase, as demonstrated by an IC_50_ value of 0.04 µg/mL (0.16 µM). This degree of inhibition is exceptionally significant, surpassing the effectiveness of kojic acid—a well-established tyrosinase inhibitor—by 88 times, with kojic acid’s IC_50_ being 2.04 µg/mL (14.36 µM). Previous studies have established that resveratrol acts both as a substrate and an inhibitor for tyrosinase, with resveratrol undergoing biotransformation by tyrosinase into an oxidized form, which is a colored product [29]. This leads to interference in the assay measurement reactions. Consequently, it is not feasible to analyze the tyrosinase inhibition using the colorimetric assay method [30].

### 2.4. Cell Viabilities and Melanogenesis

In a cell-based assay, HaCaT, HDFn, and B16F10 cells were subjected to various concentrations (0, 3.13, 6.25, 12.5, and 25 µg/mL) of *M. cochinchinensis* methanolic crude extract, as well as to 25 µg/mL of the fraction FA, the fraction FM, and the fractions C1–2 through B22, for 24 h to evaluate the impact of these extracts on cell proliferation. The findings indicated that cell viability for any treated group was consistent with the control, exhibiting 100% viability. This suggests that the extracts do not exert toxic effects on the tested cells. Furthermore, beyond the absence of toxic effects, enhanced melanogenesis inhibition in B16F10 cells was observed for 72 h in fractions containing resveratrol and oxyresveratrol at a concentration of 25 µg/mL, as detailed in Table 4. Notably, fractions Y11–Gn12, rich in resveratrol, exhibited a significant 70% reduction in melanogenesis, while fractions Gn13–B17, with high levels of oxyresveratrol, demonstrated a 40% to 60% decrease in melanogenesis. This finding underscores the potent inhibitory effect of these compounds on melanogenesis, as both resveratrol and oxyresveratrol, when used individually at a concentration of 25 µg/mL, yielded a substantial 80% inhibition of melanogenesis. The mechanism by which oxyresveratrol inhibits melanogenesis in B16F10 cells involves the suppression of tyrosinase activity and cellular oxidants [31,32,33]. Resveratrol has been demonstrated to impede α-melanocyte-stimulating hormone (α-MSH) signaling pathways in melanoma cells and to decrease the levels of tyrosinases, TRP-1, TRP-2, and microphthalmia-associated transcription factor (MITF) [34,35,36,37]. Similarly, oxyresveratrol effectively attenuates the expression of tyrosinase, TRP-1, and MITF by suppressing the expression of the genes *Tyr*, *Trp1*, and *Mitf*, subsequently lowering their levels [31,38]. The crude extract derived from *M. cochinchinensis* has been demonstrated through empirical evidence to inhibit phosphorylated protein kinase C (p-PKC) signaling pathway in Melan-A cells. The importance of the downstream signal transduction molecules in the p-PKC signaling cascade for the control of melanin production underscores the significance of this inhibition [17]. The pronounced suppression of melanin synthesis alongside the minimal cytotoxic impact displayed by *M. cochinchinensis* extracts, rich in resveratrol and oxyresveratrol, underscores their viability as secure and effective modalities for addressing hyperpigmentation issues.

### 2.5. Antioxidant Activity

In this study, *M. cochinchinensis* fractions were evaluated for antioxidant activity using the DPPH scavenging assay, and the results are presented in Table 4. Fractions FA and FM from the first-step column chromatography exhibited comparable antioxidant activities of 33.63% and 34.12%, respectively, at a concentration of 25 µg/mL. It is interesting to note that even though fraction FA contained higher amounts of resveratrol and oxyresveratrol, it exhibited almost equal antioxidant capacity compared to fraction FM. The modest antioxidant activities observed in fractions FA and FM could potentially be explained by the considerable presence of impurities within these fractions. Nonetheless, the antioxidant efficacy of fraction FA is presumably derived from its constituents of resveratrol and oxyresveratrol, while the antioxidant capability observed in fraction FM is likely attributable to morin and a variety of flavonoids and xanthones [9,16,39]. The pronounced yellowish pigment observed in the fraction FM could potentially be attributed to morin, a predominant compound in the heartwood of *M. cochinchinensis* [16], known for its array of biological properties, including antioxidant, anti-inflammatory, and antidiabetic activities [10]. Furthermore, morin has been reported to stimulate melanin synthesis in B16F10 mouse melanoma cells through the activation of MAPK signaling pathways [40].

Fractions Y11–B22 from the subsequent step exhibited a wide spectrum of antioxidant activities, with DPPH values ranging from 25.47% to 84.86% (Table 4). Notably, fractions Gn15, containing the highest concentration of oxyresveratrol, demonstrated the greatest antioxidant activity at 84.86%, which was comparable to the standard oxyresveratrol (83.68%) when applied at a concentration of 25 µg/mL. This study was specifically designed to elucidate the correlation between fractions enriched with oxyresveratrol and resveratrol and their DPPH radical-scavenging efficacy. It is important to note that the cellular antioxidant effects of oxyresveratrol and resveratrol have been extensively explored in previous research. Oxyresveratrol is known to counteract melanogenesis induced by tyrosinase-related oxidative stress in B16F10 melanoma cells [33]. Furthermore, the activation of the Nrf2/HO-1 signaling pathway by oxyresveratrol significantly enhances the ability of resveratrol to suppress UVB-induced melanin production [41], whereas resveratrol impacts keratinocytes by modulating inflammatory responses, protecting the cells from oxidative damage, and facilitating the repair of the basement membrane [42,43]. The regulatory effects of resveratrol and oxyresveratrol on melanocyte activity are underscored by their antioxidant properties, which not only hold the potential to inhibit melanin production but also provide systemic advantages in managing melasma effectively. Consequently, these compounds play a crucial role in mitigating oxidative damage, thereby attenuating premature skin aging, abnormal pigmentation, and a variety of age-related disorders, highlighting their comprehensive therapeutic efficacy [37,44].

## 3. Materials and Methods

### 3.1. Chemicals and Reagents

Acetonitrile and glacial acetic acid of HPLC grade were purchased from RCI Labscan™ (Bangkok, Thailand). Oxyresveratrol (purity ≥ 97.0%, HPLC-grade), resveratrol (purity ≥ 97.0%, HPLC-grade), L-DOPA, and kojic acid were provided by Sigma-Aldrich (St. Louis, MO, USA). All other chemical reagents were of analytical grade and used without further purification.

### 3.2. Preparation of Standard Solutions

Resveratrol and oxyresveratrol were precisely weighed and dissolved in methanol to prepare a stock solution (1 mg/mL). Standard solutions of resveratrol and oxyresveratrol were prepared through serial dilution of the stock solutions with methanol to obtain final concentrations ranging from 250 to 3.9 µg/mL. The standard solutions were filtered through a 0.45 µm filter membrane before performing HPLC analysis.

### 3.3. Plant Material, Extraction, and Isolation

The heartwoods of *M. cochinchinensis* were obtained from Chao-Krom-Poe, an herbal pharmacy in Bangkok, Thailand. The heartwood materials were shade-dried at ambient temperature and then ground into a fine powder. A preliminary study was carried out using a serial exhaustive extraction method to select a suitable extraction solvent system for the high-yield extraction of *M. cochinchinensis* heartwood. In brief, a 10 g powdered sample of *M. cochinchinensis* heartwoods was macerated at room temperature for 3 d in 100 mL of hexane, ethyl acetate, methanol, and distilled water that had been boiled for 1 h. The residues obtained after each extraction were subjected to gradual extraction using solvents of increased polarity. The residue from hexane extraction was extracted successively with ethyl acetate, methanol, and water. Meanwhile, the residue from the ethyl acetate extraction was extracted with methanol and water. On the other hand, the residues from methanol were extracted only with water. The gradual extraction using solvents of decreased polarity from water to hexane was also conducted.

The preliminary study results showed that methanol alone gave rise to the highest extraction yield of 14.6%, and it was therefore selected for the scale-up of extraction. Briefly, the *M. cochinchinensis* heartwood powder (300 g) was soaked in methanol in round bottles at a sample-to-solvent ratio of 3:10 (*w*/*v*) for 3 d at ambient temperature. The marc was re-extracted two times with the same method. The pooled extract was filtered through Whatman No. 1 filter paper (Cytiva, Marlborough, MA, USA) and then concentrated and dried at 45 °C using a rotary vacuum evaporator. After storage in a desiccator for 2 d, the samples were accurately weighed and dissolved in dimethyl sulfoxide (DMSO) to prepare stock solutions, which were then stored at –20 °C until use.

### 3.4. Mushroom Tyrosinase Inhibition Assay

The tyrosinase inhibition assay was carried out using the dopachrome method as described elsewhere, with slight modifications [30]. The crude extracts of *M. cochinchinensis* heartwood were dissolved in DMSO to a final concentration of 5 mg/mL. Meanwhile, mushroom tyrosinase (E.C. 1.14.18.1, Sigma, St. Louis, MO, USA) and L-3,4-dihydroxyphenylalanine (L-DOPA) were prepared in 20 mM phosphate buffer (PB, pH 6.8) to yield a final concentration of 100 U/mL and 2.5 mM, respectively. The reaction mixture was constituted by adding 10 μL of the extract to 20 μL of 100 U/mL mushroom tyrosinase and 130 μL of 20 mM PB (pH 6.8) in a 96-well plate. The plate was incubated at 37 °C for 10 min. After incubation, 40 μL of 2.5 mM L-DOPA was added to the reaction mixture, which was then further incubated for 10 min. Absorbance was taken at 495 nm before and after the addition of L-DOPA. For the control, DMSO was added to the reaction mixture instead of the extract. The reaction mixture without enzymes served as a blank. The percentage inhibition was calculated as follows:

$$\%inhibition=\frac{\left(∆A_{C}-∆A_{B}\right)-(∆A_{S}-∆A_{B})}{\left(∆A_{C}-∆A_{B}\right)}\times 100$$

where:

∆*A_C_* = absorbance of the control at T10 min − absorbance of the control at T0 min
∆*A_B_* = absorbance of the blank at T10 min − absorbance of the blank at T0 min
∆*A_S_* = absorbance of the extract at T10 min − absorbance of the extract at T0 min.

The inhibition ability was also expressed as the half-maximal inhibitory concentration (IC_50_). All assays were carried out in triplicate for each sample and control at different concentrations.

### 3.5. Cell Culture

Cell lines were obtained from the American Type Culture Collection (ATCC^®^, Manassas, VA, USA) and included the following: *Mus musculus* (mouse) melanoma (B16F10), human epidermal keratinocyte (HaCaT), and Primary Dermal Fibroblast Normal, Human, Neonatal (HDFn). The cells were cultured in complete Dulbecco’s Modified Eagle’s Medium (DMEM, Gibco^®^, Waltham, MA, USA) containing 10% fetal bovine serum, 25 mM glucose, 4 mM L-glutamine, 1 mM sodium pyruvate, 10 µg/mL penicillin, and 10 µg/mL streptomycin, at 37 °C, with 5% carbon dioxide (CO_2_) in a humidified atmosphere.

### 3.6. Cell Viability and Total Melanin Content Assay

Cell viability and melanin content assays were performed in quadruplicate in at least three independent experiments. Cell viability was taken as the reducing power of the living cells that reduce resazurin-based solutions (PrestoBlue™, Invitrogen, Carlsbad, CA, USA) to resorufin, which results in a bright red color and is highly fluorescent [30]. Briefly, cell lines B16F10 were cultured in a 96-well plate at a concentration of 10,000 cells/0.1 mL/well. After incubation for 24 h, an equal volume of fresh medium containing various concentrations of the test extracts or the control (0.5% DMSO) was added to the cells, and the mixture was further incubated for 24 h. Then, the cells were washed with 200 µL of 10 mM PB, followed by the addition of 100 µL of PrestoBlue™ in serum-free RMPI 1640 medium (Gibco^®^, Waltham, MA, USA) (1:10, *v*/*v*) to each well. After incubation for 1 h, the fluorescence was quantified using a microplate reader spectrofluorometer (FLUOstar^®^ Omega, BMG LABTECH, Ortenberg, Germany) at excitation and emission wavelengths of 535 and 610 nm, respectively.

The determination of the total melanin contents produced both extracellularly and intracellularly was carried out as per our previous study [30]. In brief, B16F10 cells were seeded onto a 24-well plate at an initial concentration of 50,000 cells/0.5 mL/well (DMEM, Gibco^®^, phenol red-free) and then incubated for 24 h, followed by treatment with either the test extracts or the control (0.5% DMSO) and subsequent incubation for 72 h. For the extraction of the produced melanin, a 0.5-mL volume of 2 N NaOH prepared in 20% DMSO was added directly to the cell culture medium, and the mixture was thoroughly mixed by pipetting and then incubated at ambient temperature for 1 h. Thereafter, the lysed cell extract was centrifuged at 13,000× *g* for 5 min to remove cell debris. Finally, the supernatant was transferred to a 96-well plate, and the relative melanin content was detected at 405 nm using a microplate reader. Kojic acid and oxyresveratrol were used as positive controls.

### 3.7. Stepwise Column Chromatography Fractionation and Separation of Resveratrol and Oxyresveratrol

To isolate and purify resveratrol and oxyresveratrol from the methanolic extract of *M. cochinchinensis*, a column chromatograph (10 cm × 10 cm i.d.) was filled with food-grade silica gel. The use of food-grade silica gel aimed to reduce extraction costs in this step. For the elution of resveratrol and oxyresveratrol, the column was loaded with 74.57 g of the extract and then eluted using a gradient elution system using hexane, ethyl acetate, and methanol as the solvents (Table 2). Two fractions were obtained from this separation: fractions FA and FM. The presence of resveratrol and oxyresveratrol within these fractions was subsequently determined through HPTLC analysis. As resveratrol and oxyresveratrol were found in relatively low quantities in fraction FM, this fraction was discarded, and only fraction FA was collected for further fractionation. The fractionation and separation of fraction FA were carried out in column chromatography (35 cm × 5 cm i.d.) filled with analytical-grade silica gel (pore size 60 Å, 70–230 mesh). The column was loaded with 48.4 g of the extract and subsequently eluted using a gradient system as given in Table 3. The fractions obtained were screened for the presence of resveratrol and oxyresveratrol, utilizing HPTLC analysis. Subsequently, these fractions were collected, desiccated, and stored for further investigative procedures.

### 3.8. High-Performance Thin-Layer Chromatography (HPTLC) Analysis

HPTLC separation was conducted on TLC Silica gel 60 F_254_ (20 cm × 10 cm, 200 µm thickness, Merck, Darmstadt, Germany). The standard of resveratrol and oxyresveratrol (2.0 µL at a concentration of 1000 ppm) and samples (5.0 µL at a concentration of 1000 ppm) were applied with a bandwidth of 8 mm, situated 8 mm from the lower edge, using a CAMAG^®^ Linomat 5 automatic sample spotter (CAMAG^®^, Muttenz, Switzerland). An application rate of 150 nL/s was maintained. The plate was then placed in a CAMAG^®^ Automatic Developing chamber, utilizing a mobile phase composed of toluene, ethyl acetate, and acetonitrile in a 35:15:5 ratio for a development distance of 80.0 mm. Following development, detection was carried out using a CAMAG^®^ TLC Visualizer 3 at wavelengths of 254 nm, 366 nm, and under white light. Densitometric scanning occurred at 366 nm with a CAMAG^®^ TLC Scanner 4. The scanning slit dimensions were set to 6.00 mm × 0.45 mm, with a scanning speed of 20 mm/s. Densitograms were analyzed using visionCATS (version 3.0) software.

### 3.9. LC-MS/MS and HPLC Analyses

Detection of the compounds resveratrol and oxyresveratrol was performed on a Shimadzu LCMS-8030 HPLC system coupled to a quadrupole tandem mass spectrometer (Shimadzu Corporation, Kyoto, Japan). Separation was achieved on an InertSustain^®^ C18 column (2.1 mm i.d. × 150 mm, 3 µm) (GL Sciences Inc., Tokyo, Japan) coupled with an InertSustain^®^ C18 guard column 2.1 mm i.d. × 10 mm, 3 µm), with a column temperature of 30 °C. The mobile phase comprised deionized water (A) and acetonitrile (B). The elution was carried out in gradient mode (0–1 min, 50% B; 1–2 min, 50–70% B; 2–5 min, 90% B; 5–7 min, 90% B; 9–15 min, 50% B) at a flow rate of 0.2 mL/min. The mass spectrometric conditions were as follows: nebulizer gas (N_2_) flow rate, 3 L/min; drying gas (N_2_) flow rate, 15 L/min; interface temperature, 350 °C; heat block temperature, 400 °C; desolvation line temperature, 250 °C; detector voltage, 4.5 kV. The full mass scan spectra were recorded in the negative ionization mode, in the *m*/*z* range of 200–1200. The instrument control and data acquisition were performed using the LabSolutions 5.42v software (Shimadzu). The results of the auto-optimizations are summarized in Table 5.

The contents of resveratrol and oxyresveratrol were determined by HPLC. The HPLC system (Agilent 1290 Infinity, Agilent Technologies, Santa Clara, CA, USA) consisted of a binary pump (G4220A), diode array detector (G4212A), and autosampler (G4226A). The separation was performed on a Zorbax^®^ SB-C18 rapid resolution HD column (Agilent, 2.1 mm i.d. × 150 mm, 1.8 µm) coupled with a Zorbax^®^ SB-C18 guard column (2.1 mm i.d. × 5 mm, 1.8 µm). The flow rate was 0.2 mL/min, and the injection volume was 5 µL. The detection wavelength was set at 320 nm, and the column temperature was maintained at 40 °C. The mobile phase comprised acetonitrile (A) and 0.5% acetic acid in water (B). The following gradient solvent system was applied: 0–10 min, 18–25% A; 10–25 min, 25–30% A; 25–27 min, 30–40% A; 27–30 min, 40–90% A. The data were collected and analyzed with 2D-LC software (version A.01.04).

### 3.10. DPPH Free Radical Scavenging Activity

The DPPH-scavenging assay was carried out according to [45]. In brief, a 5 µL volume of each fraction (1000 µg/mL in DMSO) obtained after the column chromatography fractionation of the *M. cochinchinensis* extract was reacted with 195 μL of 100 µM DPPH methanolic solution in a 96-well microplate. Five microliters of DMSO were used as the control. The plate was then incubated at 37 °C for 30 min, and the absorbance was then measured at 515 nm (FLUOstar^®^ Omega, BMG LABTECH, Germany). The DPPH radical-reducing activity of the fraction was calculated as follows:

% scavenging activity = [(A_0_ − A_1_)/A^0^] × 100

where A_0_ is the absorbance of the control reaction, and A_1_ is the absorbance in the presence of the tested fraction.

## 4. Conclusions

This investigation has demonstrated that the heartwood extract of *Maclura cochinchinensis* is notably rich in resveratrol and oxyresveratrol. These compounds significantly outperform kojic acid in inhibiting tyrosinase activity and melanogenesis, suggesting their utility as effective natural alternatives for skin lightening. To ensure consistency and quality of the extract, resveratrol and oxyresveratrol can serve as quality markers for standardizing the extract, with minimum concentrations set at 4 mg/g and 30 mg/g, respectively. For cosmetic applications, a partial purification of the extract is advised, utilizing column chromatography with food-grade silica gels and a hexane-ethyl acetate solvent system. This process separates the extract into yellow fractions (FA) enriched with resveratrol and oxyresveratrol, in contrast to dark brown fractions (FM) obtained with methanol, which, despite lacking whitening properties, exhibit antioxidant potential due to their phenolic content. Cytotoxicity assays conducted on HaCaT, HDFn, and B16F10 cells indicate that the crude heartwood extract and all chromatographic fractions are non-toxic at concentrations up to 25 µg/mL, underscoring their potential safety for use in skin-lightening formulations. Consequently, it is advised to subject the *M. cochinchinensis* heartwood extract to partial purification by column chromatography to eliminate the dark brown fractions prior to their incorporation into cosmetic formulations, thereby optimizing the skin-whitening efficacy of the extract and ensuring safety.

## 5. Patents

A petty patent application was filed with the Department of Intellectual Property in Thailand, under Application Number 2203001856.

## Figures and Tables

**Table 1:** Extraction yields and anti-tyrosinase activities of the crude extracts of *M. cochinchinensis*.

No.	Solvents Used in Serial Exhaustive Extraction *	Extraction Yield		Tyrosinase Inhibitory Activity	
		mg **	%	% ***	IC_50_ (µg/mL) ****
1	Hexane (H_0_)	0	0	ND	ND
	Ethyl acetate (H_1_)	240	2.4	69.98	0.52 ± 0.04
	Methanol (H_2_)	900	9.0	83.64	0.28 ± 0.11
	Water (H_3_)	640	6.4	60.65	0.95 ± 0.12
2	Ethyl acetate (A_0_)	350	3.5	74.31	0.71 ± 0.18
	Methanol (A_1_)	940	9.4	82.50	0.12 ± 0.01
	Water (A_2_)	300	3.0	61.81	2.36 ± 0.18
3	Methanol (M_0_)	1460	14.6	80.61	0.21 ± 0.01
	Water (M_1_)	180	1.8	27.11	6.90 ± 1.03
4	Water (W_0_)	860	8.6	69.32	0.67 ± 0.03
	Methanol (W_1_)	660	6.6	78.53	0.19 ± 0.09
	Ethyl acetate (W_2_)	200	2.0	79.30	0.38 ± 0.08
	Hexane (W_3_)	240	2.4	ND	ND
	Oxyresveratrol	-	-	98.48	0.020 ± 0.004
	Kojic acid	-	-	18.81	13.85 ± 1.52

* The powdered sample of 10 g was used for each serial exhaustive extraction group. ** mg per 10 g of the powdered sample, the raw material. *** The concentration of the extract applied was 2.5 µg/mL. **** IC50—Half maximal inhibitory concentration. ND—Not detected.

**Table 2:** The nonpolar-to-polar solvent system and food-grade silica gel used in the first step of column chromatography for the separation of the methanolic extract of *M. cochinchinensis*.

No.	Fractions Collected	Eluent Ratio (%)			Elution Volume (mL)
		Hexane	Ethyl Acetate	Methanol	
1	FA	100	-	-	800
		80	20	-	1600
		60	40	-	2400
		-	100	-	1600
2	FM	-	-	100	1000

**Table 3:** The nonpolar-to-polar solvent system was used for further purification of the fraction FA collected from the previous column chromatographic separation.

No.	Fractions Collected *	Eluent Ratio (%)			Elution Volume (mL)
		Hexane	Ethyl Acetate	Methanol	
1	C1–2	100	-	-	1400
2	C3–Y10	80	20	-	2100
3	Y11–Gn13	60	40	-	2100
4	Gn14	55	45	-	300
5	Gn15	50	50	-	300
6	Gn16–B18	40	60	-	700
7	B19–B20	20	80	-	700
8	B21	-	100	-	700
9	B22	-	-	100	1400

* C—colorless solution, Y—yellow solution, Gn—yellowish-green solution, B—brown solution.

**Table 4:** Quantitative yields and HPLC analysis of resveratrol and oxyresveratrol in methanolic extracts from *M. cochinchinensis* heartwood and various fractions, elution profiles using analytical-grade silica gel, and DPPH radical scavenging activities, anti-tyrosinase, and melanogenesis.

Fraction	Yield (g)	Resveratrol		Oxyresveratrol		DPPH Scavenging Activity (%) *^,^**	Anti-Mushroom Tyrosinase, IC_50_ (µg/mL)	Melanogenesis in B16F10 (%) *^,^***^,†^
		Conc. (mg/g)	Total (mg)	Conc. (mg/g)	Total (mg)			
Methanolic crude extract	74.46	4.32	321.67	33.62	2503.35	41.76 ± 3.64	0.30 ± 0.02	50.62 ± 1.26
FA	48.46	6.45	312.57	50.39	2441.90	33.63 ± 9.78	0.25 ± 0.01	45.59 ± 2.02
FM	13.68	0.13	1.78	3.82	52.26	34.12 ± 2.55	NF	105.65 ± 7.66
C1–2	0.23	ND	ND	NF	NF	NF	NF	112.09 ± 6.67
C3	0.11	ND	ND	NF	NF	NF	NF	109.02 ± 8.13
C4	0.174	ND	ND	NF	NF	NF	NF	105.00 ± 6.07
Gn5	0.13	ND	ND	NF	NF	NF	NF	106.53 ± 7.42
Gn6	0.09	ND	ND	NF	NF	NF	NF	94.71 ± 2.31
Y7	0.15	0.04	0.006	NF	NF	NF	NF	103.43 ± 7.98
Y8	0.22	0.05	0.011	NF	NF	NF	NF	33.50 ± 2.33
Y9	0.31	0.07	0.02	NF	NF	29.37 ± 2.16	NF	34.80 ± 3.24
Y10	0.22	0.22	0.05	NF	NF	46.76 ± 5.12	0.27 ± 0.02	38.32 ± 2.69
Y11	1.21	94.90	114.83	NF	NF	44.21 ± 6.34	0.21 ± 0.03	29.45 ± 1.54
Gn12	0.35	110.21	38.57	0.16	0.06	46.22 ± 5.97	0.13 ± 0.01	30.63 ± 4.65
Gn13	1.78	63.85	113.65	34.86	62.05	56.66 ± 7.01	0.10 ± 0.00	47.40 ± 3.21
Gn14	0.82	1.23	1.01	195.62	160.41	77.67 ± 8.82	0.05 ± 0.01	45.30 ± 5.37
Gn15	3.35	NF	NF	321.93	1078.47	84.86 ± 8.97	0.05 ± 0.00	46.72 ± 6.62
Gn16	0.99	NF	NF	274.59	271.84	75.69 ± 6.54	0.12 ± 0.02	47.00 ± 5.12
B17	1.31	NF	NF	88.96	116.54	68.95 ± 7.32	0.30 ± 0.02	66.73 ± 5.64
B18	3.42	NF	NF	2.28	7.80	35.46 ± 2.11	0.32 ± 0.06	91.81 ± 7.72
B19	8.49	NF	NF	1.81	15.37	27.80 ± 1.01	NF	90.78 ± 6.13
B20	3.07	NF	NF	NF	NF	31.35 ± 2.78	NF	91.51 ± 8.11
B21	4.68	NF	NF	NF	NF	31.28 ± 2.24	NF	96.10 ± 5.78
B22	0.33	NF	NF	NF	NF	25.47 ± 1.09	NF	93.00 ± 6.54
Resveratrol						40.74 ± 2.56	ND	20.78 ± 1.24
Oxyresveratrol						83.68 ± 6.13	0.04 ± 0.00	20.67 ± 1.17
Kojic acid						NF	2.04 ± 0.10	68.45 ± 8.34

* The final concentration of the extract or the fraction applied was 25 µg/mL. ** 2.5% DMSO served as the control. *** 0.5% DMSO served as the control. **^†^** Cell viabilities were found to be comparable to the control, exhibiting 100% viability at 72 h. ND—not determined; NF—not found.

**Table 5:** LC-MS/MS analysis conditions for resveratrol and oxyresveratrol detection.

No.	Substances	Retention Time (min)	Precursor Ion [M − H]^−^ (*m*/*z*)	Product Ion (*m*/*z*)	Q1 Pre Bias (V)	Collision Energy (V)	Q3 Pre Bias (V)
1	Resveratrol	4.369	227.05	181.1	27.0	11.0	24.0
2	Oxyresveratrol	2.753	243.10	175.2	28.0	13.0	12.0

## Data Availability

Data are contained within the article.
